# A 6-mRNA host response classifier in whole blood predicts outcomes in COVID-19 and other acute viral infections

**DOI:** 10.1038/s41598-021-04509-9

**Published:** 2022-01-18

**Authors:** Ljubomir Buturovic, Hong Zheng, Benjamin Tang, Kevin Lai, Win Sen Kuan, Mark Gillett, Rahul Santram, Maryam Shojaei, Raquel Almansa, Jose Ángel Nieto, Sonsoles Muñoz, Carmen Herrero, Nikolaos Antonakos, Panayiotis Koufargyris, Marina Kontogiorgi, Georgia Damoraki, Oliver Liesenfeld, James Wacker, Uros Midic, Roland Luethy, David Rawling, Melissa Remmel, Sabrina Coyle, Yiran E. Liu, Aditya M. Rao, Denis Dermadi, Jiaying Toh, Lara Murphy Jones, Michele Donato, Purvesh Khatri, Evangelos J. Giamarellos-Bourboulis, Timothy E. Sweeney

**Affiliations:** 1 Inflammatix, Inc., 863 Mitten Rd, Suite 104, Burlingame, CA 94010 USA; 2 Institute for Immunity, Transplantation and Infection, School of Medicine, Stanford University, Palo Alto, CA 94305 USA; 3 Center for Biomedical Informatics Research, Department of Medicine, Stanford University, Stanford, CA 94305 USA; 4 Centre for Immunology and Allergy Research, Westmead Institute for Medical Research, Sydney, Australia; 5 Department of Intensive Care Medicine, Nepean Hospital, Sydney, Australia; 6 Nepean Genomic Research Group, Nepean Clinical School, University of Sydney, Sydney, Australia; 7 Marie Bashir Institute for Infectious Diseases and Biosecurity, Sydney, Australia; 8 Department of Emergency Medicine, Westmead Hospital, Sydney, Australia; 9 Department of Emergency Medicine, National University Hospital Singapore, Singapore, Singapore; 10 Department of Emergency Medicine, Royal North Shore Hospital, Sydney, Australia; 11 Department of Emergency Medicine, St Vincent Hospital, Sydney, Australia; 12 Group for Biomedical Research in Sepsis (BioSepsis), Instituto de Investigación Biomédica de Salamanca (IBSAL), Salamanca, Spain; 13 Hospital Universitario Río Hortega, Valladolid, Spain; 14 Servicio de Urgencias de Atención Primaria, Salamanca, Spain; 15 4th Department of Internal Medicine, National and Kapodistrian University of Athens, Medical School, 124 62 Athens, Greece; 16 Cancer Biology Program, Stanford University, Stanford, CA 94305 USA; 17 Immunology Program, Stanford University, Stanford, CA 94305 USA; 18 Division of Critical Care Medicine, Department of Pediatrics, School of Medicine, Stanford University, Stanford, CA 94305 USA

**Keywords:** Infectious diseases, Prognostic markers, Molecular medicine, Viral infection, Machine learning, Statistical methods

## Abstract

Predicting the severity of COVID-19 remains an unmet medical need. Our objective was to develop a blood-based host-gene-expression classifier for the severity of viral infections and validate it in independent data, including COVID-19. We developed a logistic regression-based classifier for the severity of viral infections and validated it in multiple viral infection settings including COVID-19. We used training data (N = 705) from 21 retrospective transcriptomic clinical studies of influenza and other viral illnesses looking at a preselected panel of host immune response messenger RNAs. We selected 6 host RNAs and trained logistic regression classifier with a cross-validation area under curve of 0.90 for predicting 30-day mortality in viral illnesses. Next, in 1417 samples across 21 independent retrospective cohorts the locked 6-RNA classifier had an area under curve of 0.94 for discriminating patients with severe vs. non-severe infection. Next, in independent cohorts of prospectively (N = 97) and retrospectively (N = 100) enrolled patients with confirmed COVID-19, the classifier had an area under curve of 0.89 and 0.87, respectively, for identifying patients with severe respiratory failure or 30-day mortality. Finally, we developed a loop-mediated isothermal gene expression assay for the 6-messenger-RNA panel to facilitate implementation as a rapid assay. With further study, the classifier could assist in the risk assessment of COVID-19 and other acute viral infections patients to determine severity and level of care, thereby improving patient management and reducing healthcare burden.

## Introduction

The emergence of the SARS-coronavirus 2 (SARS-CoV-2), causative agent of COVID-19, and its rapid pandemic spread has led to a global health crisis with more than 54 million cases and more than 1 million deaths to date^1^. COVID-19 presents with a spectrum of clinical phenotypes, with most patients exhibiting mild-to-moderate symptoms, and 20% progressing to severe or critical disease, typically within a week^2–6^*.* Severe cases are often characterized by acute respiratory failure requiring mechanical ventilation and sometimes progressing to ARDS and death^7^. Illness severity and development of ARDS are associated with older age and underlying medical conditions^3^.

Yet, despite the rapid progress in developing diagnostics for SARS-CoV-2 infection, existing prognostic markers ranging from clinical data to biomarkers and immunopathological findings have proven unable to identify which patients are likely to progress to severe disease^8^. Poor risk stratification means that front-line providers may be unable to determine which patients might be safe to quarantine and convalesce at home, and which need close monitoring. Early identification of severity along with monitoring of immune status may also prove important for selection of treatments such as corticosteroids, intravenous immunoglobulin, or selective cytokine blockade^9–11^.

A host of lab values, including neutrophilia, lymphocyte counts, CD3 and CD4 T-cell counts, interleukin-6 and -8, lactate dehydrogenase, D-dimer, AST, prealbumin, creatinine, glucose, low-density lipoprotein, serum ferritin, and prothrombin time rather than viral factors have been associated with higher risk of severe disease and ARDS^3,12,13^. While combining multiple weak markers through machine learning (ML) has a potential to increase test discrimination and clinical utility, applications of ML to date have led to serious overfitting and lack of clinical adoption^14^. The failure of such models arises both from a lack of clinical heterogeneity in training, and from the pragmatic nature of the variable selection, which uses existing lab tests which may not be ideal for the task. Furthermore, a number of the lab markers are late indicators of severity since by the time they become abnormal, patient is already very sick.

The host immune response represented in the whole blood transcriptome has been repeatedly shown to diagnose presence, type, and severity of infections^15–19^. By leveraging clinical, biological, and technical heterogeneity across multiple independent datasets, we have previously identified a conserved host response to respiratory viral infections^16^ that is distinct from bacterial infections^15–17^ and can identify asymptomatic infection. Recently, we have demonstrated that this conserved host response to viral infections is strongly associated with severity of outcome, including in patients infected with SARS-CoV-2, chikungunya, and Ebola^20^. We have also demonstrated that conserved host immune response to infection can be an accurate prognostic marker of risk of 30-day mortality in patients with infectious diseases^18^. Most importantly, we have demonstrated that accounting for biological, clinical, and technical heterogeneity identifies more generalizable robust host response-based signatures that can be rapidly translated on a targeted platform^19^.

Based on these previous results that there is a shared blood host-immune response-based mRNA prognostic signature among patients with acute viral infections, we hypothesized that a parsimonious, clinically translatable gene signature for predicting outcome in patients with viral infection can be identified. We tested this hypothesis by integrating 21 independent data sets with 705 peripheral blood transcriptome profiles from patients with acute viral infections and identified a 6-mRNA host-response-based signature for mortality prediction across these multiple viral datasets. Next, we validated the locked model in another 21 independent retrospective cohorts of 1417 blood transcriptome profiles of patients with a variety of viral infections (non-COVID). Finally, we validated our 6-mRNA model in independent prospectively and retrospectively collected cohorts of patients with COVID-19, demonstrating its ability to predict outcomes despite having been entirely trained using non-COVID data. Our results suggest the conserved host response to acute viral infection can be used to predict its outcome. Finally, we showed validity of a rapid isothermal version of the 6-mRNA host-response-signature which is being further developed into a rapid molecular test (CoVerity™) to assist in improving management of patients with COVID-19 and other acute viral infections.

## Materials and methods

### Data collection, curation, and sample labeling

**Figure 1:**
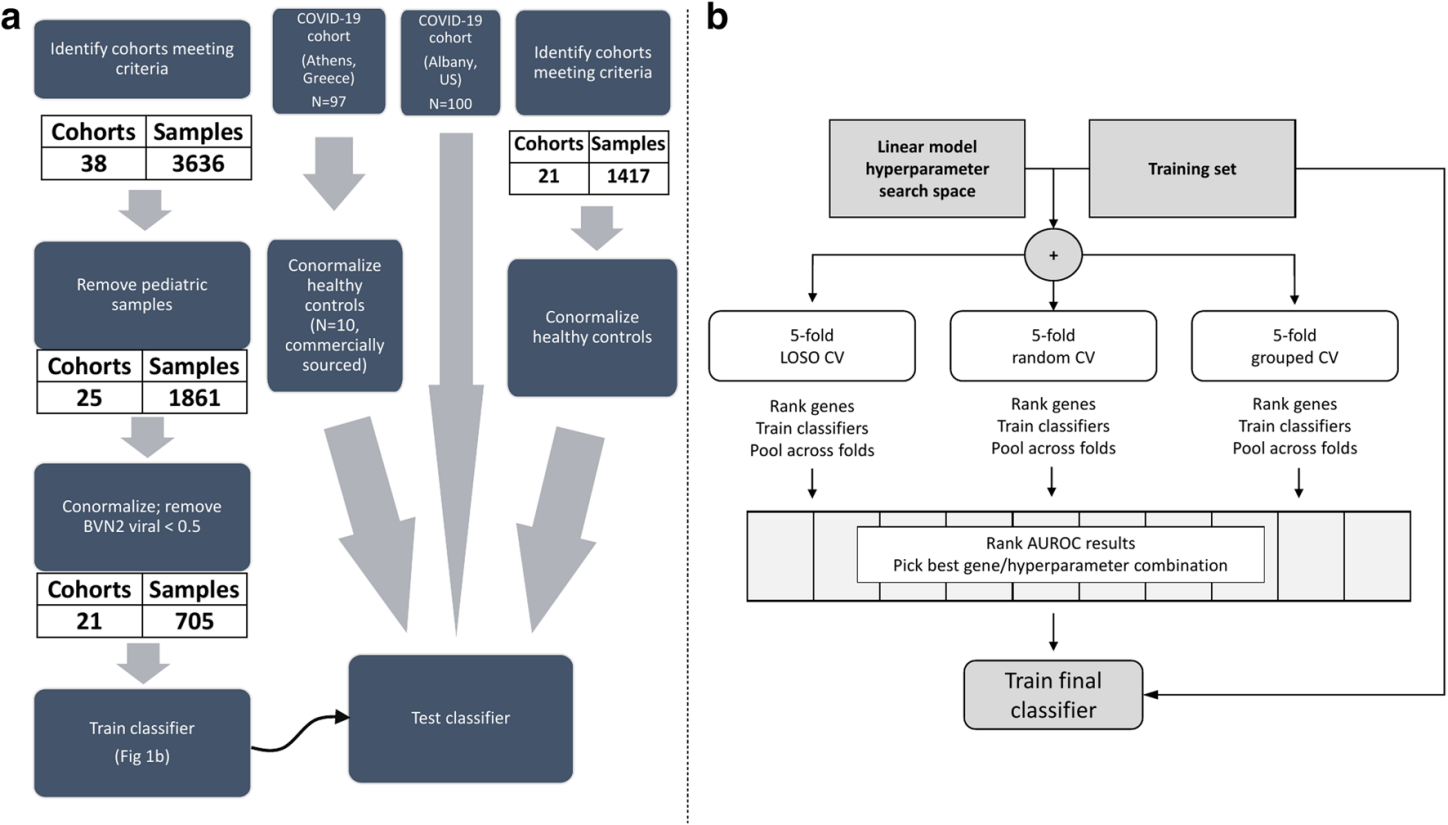
Study flow. (**a**) Clinical data flows for training and testing. (**b**) Machine learning worfklow used to develop and validate the 6-mRNA viral severity classifier. *LOSO* Leave-One-Study-Out, *CV* cross-validation, *AUROC* Area Under ROC curve.

We searched public repositories (National Center for Biotechnology Information Gene Expression Omnibus and European Bioinformatics Institute ArrayExpress) for studies of typical acute infection with mortality data present. After removal of pediatric and entirely non-viral datasets, we identified 17 microarray, or RNAseq peripheral blood acute infection studies composed of samples from 1861 adult patients with either 28-day or 30-day mortality information as “training samples” (Fig. 1 and Table 1). We processed and co-normalized these datasets as described previously^19^. In addition to this public microarray/RNAseq data, we included 394 samples across 4 independent cohorts^19^ that were profiled using NanoString nCounter, of which 14 patients with viral infection had died (Table 1).

**Table 1:** Characteristics of viral infection studies used for training. *COPD* chronic pulmonary obstruction disorder, *ICU* intensive care unit, *TB* tuberculosis, *CAP* community-acquired pneumonia.

Study identifier	First author or PI	Study description	Timing of sample collection	N (survivors/non-survivors)	Age (median, IQR)	Male (n (%))	Country	Platform
E-MEXP-3589	Almansa	Patients hospitalized with COPD exacerbation	Hospital/ICU admission	5 (5/0)	Unk	5(100)	Spain	Agilent
E-MTAB-1548	Almansa	Surgical patients with sepsis (EXPRESS)	Average post-operation day 4	3 (3/0)	78.0 (71.5–79.5)	3(100)	Spain	Agilent
E-MEXP-3162	Van de Weg	Uncomplicated dengue	Within 48 h of onset	21 (21/0)	Unk	Unk	Indonesia	Affymetrix
GSE13015 (GPL6102)	Pankla	Sepsis, many cases from burkholderia	Within 48 h of diagnosis	3 (2/1)	54.0 (46.0–55.5)	1(33)	Thailand	Illumina
GSE13015 (GPL6947)				2 (2/0)	64.5 (56.2–72.8)	1(50)		Illumina
GSE21802	Bermejo-Martin	Pandemic H1N1 in ICU	Within 48 h of ICU admission	6 (5/1)	Unk	Unk	Canada	Illumina
GSE22098	Berry	Patients with active TB and other inflammatory and infectious diseases	At admission	39 (39/0)	31.0 (19.0–47.0)	6(15)	UK, South Africa	Illumina
GSE27131	Berdal	Severe H1N1 influenza	Admission to ICU	3 (2/1)	38.0 (31.5–46.0)	3(100)	Norway	Affymetrix
GSE28991	Naim	Acute dengue fever	Within 72 h of onset	11 (11/0)	Unk	Unk	Singapore	Illumina
GSE32707	Dolinay	Critically ill patients in ICU (Sepsis, SIRS and/or ARDS)	Admission to ICU	7 (5/2)	45.0 (39.0–50.5)	4(57)	USA	Illumina
GSE40012	Parnell	Bacterial or influenza A pneumonia or SIRS	Admission to ICU	11 (11/0)	Unk	4(36)	Australia	Illumina
GSE54514	Parnell	Sepsis patients in ICU	Admission to ICU	2 (2/0)	62.5 (60.2–64.8)	1(50)	Australia	Illumina
GSE51808	Kwissa	Acute dengue fever	1–8 days after onset	28 (28/0)		Unk	Thailand	Affymetrix
GSE60244	Suarez	Lower respiratory tract infections	Within 24 h of admission	62 (62/0)	59.0 (50.0–74.5)	24(39)	USA	Illumina
GSE65682	Scicluna	Suspected but negative for CAP	Within 24 h of ICU admission	9 (7/2)	67.0 (63.0–73.0)	7(78)	Netherlands	Affymetrix
GSE68310	Zhai	Outpatients with acute respiratory viral infections	Within 48 h of onset	75 (75/0)	21.0 (20.4–22.3)	34(45)	USA	Illumina
GSE82050	Tang	Moderate and severe influenza infection	Within 24 h of admission	17 (17/0)	55.0 (45.0–72.0)	Unk	Germany	Agilent
GSE95233	Venet	Septic shock patients in ICU	Admission to ICU	7 (5/2)	47.0 (42.0–65.0)	5(71)	France	Affymetrix
Australia/WIMR	Tang	Community or hospital clinics with influenza-like illness	At presentation	332 (321/11)	48.0 (32.0–63.5)	129(39)	Australia	Nanostring
Stanford ICU databank	Rogers	Suspected sepsis with ARDS risk factors	Admission to ICU	8 (6/2)	62.0 (55.5–67.2)	4(50)	USA	Nanostring
PROMPT	Giamarellos-Bourboulis	Suspected infection with 2 + SIRS	Admission to ED	1 (1/0)	78.0	0(0)	Greece	Nanostring
PREVISE	Herrero	Outpatient urgent care with suspected CAP	At presentation	53 (52/1)	78.0 (66.0–87.0)	33(62)	Spain	Nanostring

The number of cases with clinically adjudicated viral infection and known mortality outcome among the training samples was too low for robust modeling. Thus, to increase the number of training samples, we adopted the following approach. We split the samples with known mortality outcome in two groups: (1) patients with clinically adjudicated viral infections and known severity outcome (Group 1) and (2) patients with clinically adjudicated infection, but unknown infection type, and known severity outcome (Group 2). Group 1 was used as is. Unfortunately, it was too small for robust classifier development. Although Group 2 was larger, it could not be used directly because we did not know the infection type. To circumvent this problem, we assigned viral infection status using a previously developed gene-expression-based bacterial/viral classifier, whose accuracy approaches that of clinical adjudication. Specifically, we utilized an updated version of our previously described neural network-based classifier for diagnosis of bacterial vs. viral infections called ‘Inflammatix Bacterial-Viral Noninfected version 2’ (IMX-BVN-2)^18^. We note that we did not apply IMX-BVN-2 to any samples that were already diagnosed to have bacterial infection, i.e., we did not reclassify any bacterial infection samples as viral infections. The rationale is that this method would increase the number of mortality samples with viral infection, without introducing many false positives. In essence, IMX-BVN-2 classifier score was used as a substitute for the (unavailable) clinical adjudication of the infection status in Group 2. For all samples, we applied IMX-BVN-2 to assign a probability of bacterial or viral infection and retained samples for which viral probability according to IMX-BVN-2 was ≥ 0.5. We refer to this assessment of viral infection as computer-aided adjudication. Out of 1861 samples, we found 311 samples which had IMX-BVN-2 probability of viral infection ≥ 0.5, of which 9 patients died within 30-day period. Thus, overall, we included 705 blood samples across 21 independent studies from patients with computer aided adjudication of viral infection and short-term mortality outcome. Importantly, none of these patients had SARS-CoV-2 infection as they were all enrolled prior to November 2019.

### Selection of variables for classifier development

We preselected 29 mRNAs from which to develop the classifier for several biological and practical reasons. Biologically, the 29 mRNAs are composed of an 11-gene set for predicting 30-day mortality in critically ill patients and a repeatedly validated 18-gene set that can identify viral vs bacterial or noninfectious inflammation^17–19^. Thus, we hypothesized that if a generalizable viral severity signature were possible, we likely had appropriate (and pre-vetted) variables here. By limiting our input variables, we also lowered our risk of overfitting to the training data. From a practical perspective, first, we are developing a point-of-care diagnostic platform for measuring these 29 genes in less than 30 min. Hence, a classifier developed using a subset of these 29 genes would allow us to develop a rapid point-of-care test on our existing platform. Second, 4 of the 21 cohorts included in the training were Inflammatix studies that profiled these 29 genes using NanoString nCounter and therefore for those studies this was the only mRNA expression data available.

### Development of a classifier using machine learning

We analyzed the 705 viral samples using cross-validation (CV) for ranking and selecting machine learning classifiers. We explored three variants of cross-validation: (1) fivefold random CV, (2) fivefold grouped CV, where each fold comprises multiple studies, and each study is assigned to exactly one CV fold, and (3) leave-one-study-out (LOSO), where each study forms a CV fold. We included non-random CV variants because we recently demonstrated that the leave-one-study-out cross-validation may reduce overfitting during training and produce more robust classifiers, for certain datasets^19^. The hyperparameter search space was based on machine learning best practices and our previous results in model optimization in infectious disease diagnostics^21^. We compared multi-layer perceptron neural network, logistic regression classifier, Light Gradient Boosting Machine, XGBoost, Support Vector Machine and Random Forest. For rapid turnaround we limited the number of hyperparameter configurations we searched to 1000 per classifier. Finally, we applied forward selection and univariate feature ranking to select six genes for translation to a rapid molecular assay. We followed best practices to avoid overfitting in the gene selection process^22,23^.

We performed cross-validations for each of the hyperparameter configurations. Within each fold, we sorted the absolute value of the genes’ Pearson correlation with class label (survived/died). We then trained a classifier using the six top-ranked genes and applied it to the left-out fold. The predicted probabilities from the folds were pooled, and the Area Under a Receiver Operating Characteristic (AUROC) curve over the pooled cross-validation probabilities was used as a metric to rank classification models. The final ranking of genes was determined using average ranking across the CV folds. Once the best-ranking model hyperparameters were selected and the final list of six genes was established, the final model was trained using the entire training set and the ‘locked’ hyperparameters. The corresponding model weights were locked, and the final classifier was then tested in an independent prospective cohort of patients with COVID-19, and in independent retrospective cohort of patients with viral infections without COVID-19.

To assess the impact of the computer-aided adjudication, we also built model based on clinical adjudications only. The two models were assessed using the independent validation data. This was performed after the primary analyses to maintain independence of the validation data.

### Retrospective non-COVID-19 patient cohort for validation of 6-mRNA classifier

We selected a subset of samples from our previously described database of 34 independent cohorts derived from whole blood or peripheral blood mononuclear cells (PBMCs)^20^. From this database we removed all samples that were used in our analysis for identifying the 6-gene signature, leaving 1417 samples across 21 independent cohorts for validation of the 6-mRNA classifier (Supplementary Table 1). The samples in these datasets represented the biological and clinical heterogeneity observed in the real-world patient population, including healthy controls and patients infected with 16 different viruses with severity ranging from asymptomatic to fatal viral infection over a broad age range (< 12 months to 73 years) (Fig. 1a and Supplementary Table 1). Notably, the samples were from patients enrolled across 10 different countries representing diverse genetic backgrounds of patients and viruses. Finally, we included technical heterogeneity in our analysis as these datasets were profiled using microarrays from different manufacturers.

We renormalized all microarray datasets using standard methods when raw data were available from the Gene Expression Omnibus database. We applied Guanine Cytosine Robust Multiarray Average (gcRMA) to arrays with mismatch probes for Affymetrix arrays. We used normal-exponential background correction followed by quantile normalization for Illumina, Agilent, GE, and other commercial arrays. We did not renormalize custom arrays and used preprocessed data as made publicly available by the study authors. We mapped microarray probes in each dataset to Entrez Gene identifiers (IDs) to facilitate integrated analysis. If a probe matched more than one gene, we expanded the expression data for that probe to add one record for each gene. When multiple probes mapped to the same gene within a dataset, we applied a fixed-effect model. Within a dataset, cohorts assayed with different microarray types were treated as independent.

### Standardized severity assignment for retrospective non-COVID-19 patient samples

We used standardized severity for each of the 1417 samples as described before by Zheng et al.^20^*.* First, we assigned the same phenotype (uninfected or infected) to each sample as described in the original publication. Next, we assigned one out of seven standardized severity categories to each sample based on the corresponding cohort description in the original publication. The seven standardized categories are healthy, asymptomatic, mild, moderate, severe, critical, and fatal. Each severity category is defined as follows. Healthy controls are individuals who were asymptomatic and did not have any infection or any known comorbidities. The samples assigned asymptomatic were either from afebrile asymptomatic individuals who were positive for virus or had fully recovered from a viral infection with completely resolved symptoms. We assigned mild category to samples from symptomatic individuals, who tested positive for viral infection, that were either managed as outpatient or discharged from the emergency department (ED). The samples assigned to the moderate category included symptomatic individuals with viral infection who were admitted to the general ward but did not require supplemental oxygen. The samples in the serious category included those from symptomatic individuals with viral infection who were admitted to general ward with supplemental oxygen or admitted to the intensive care unit (ICU) without requiring mechanical ventilation or inotropic support. We assigned the samples from symptomatic individuals with viral infection who were on mechanical ventilation in the ICU or were diagnosed with acute respiratory distress syndrome (ARDS), septic shock, or multiorgan dysfunction syndrome to the critical category. Finally, the samples from patients with viral infection who died in the ICU were assigned to the fatal category.

Several datasets (GSE101702, GSE38900, GSE103842, GSE66099, GSE77087) did not provide sample-level severity data. We used the following rules to assign severity category to each sample in these datasets. We categorized all samples in the dataset as “moderate”, if most of the patients (> 70%) were admitted to the general ward, or only a subset of patients (< 20%) admitted to the general ward required supplemental oxygen. We assigned all samples in a dataset to the “severe” category if most (> 20%) of patients had either been admitted to the general ward and categorized as “severe” by original authors, or required supplemental oxygen or ICU admission without mechanical ventilation.

The samples in this group were further categorized on the basis of when blood sampling was performed relative to severe disease classification. If blood samples for a study were taken earlier in the course of disease, when prognostic severity classifier would be more clinically useful, it was labeled “early” study; conversely, if sampling was done late in the course of the disease, when clinical severity was already obvious, the study was labeled “late”. The clinical utility of “late” sampling is limited because no testing is needed at this stage. Based on review of available literature associated with the studies in the retrospective non-COVID-19 dataset, we conservatively estimated that up to about 35% of patients were sampled “late”; and the remaining patients were sampled “early”. In particular, to ensure our estimates are upper bound of the performance impact due to the “late” studies, sampling on admission to ICU was treated as “late”.

### Retrospective COVID-19 patient cohort

We used COVID-19 samples (N = 100) from GSE157103^24^. Briefly, the dataset includes RNA-Seq expression data for adult patients hospitalized for suspected COVID-19 in April 2020, in Albany Medical Center (Albany, NY, USA). We applied the 6-mRNA classifier to the gene expression data for participants who tested positive for COVID-19. The expression values were estimated by “RNA-Seq by Expectation Minimization” algorithm by the study authors. We used the binary “mechanical-ventilation” status (yes/no) provided by authors as indicator of the disease severity.

### Prospective COVID-19 patient cohort

Blood samples were collected between March and April 2020 from three study sites participating in the Hellenic Sepsis Study Group (http://www.sepsis.gr). The studies were conducted following approvals for the collection of biomaterial for transcriptomic analysis for patients with lower respiratory tract infections provided by the Ethics Committees of the participating hospitals. All methods were performed in accordance with the relevant guidelines and regulations. Participants were adults with written informed consent provided by themselves or by first-degree relatives in the case of patients unable to consent, with molecular detection of SARS-CoV-2 in respiratory secretions and radiological evidence of lower respiratory tract involvement. PAXgene^®^ Blood RNA tubes were drawn within the first 24 h from admission along with other standard laboratory parameters. Data collection included demographic information, clinical scores (SOFA, APACHE II), laboratory results, length of stay and clinical outcomes. Patients were followed up daily for 30 days; severe disease was defined as respiratory failure (PaO_2_/FiO_2_ ratio less than 150 requiring mechanical ventilation) or death. PAXgene Blood RNA samples were shipped to Inflammatix, where RNA was extracted and processed using NanoString nCounter^®^, as previously described^19^. The 6-mRNA scores were calculated after locking the classifier weights.

### Healthy controls

We acquired five whole blood samples from healthy controls through a commercial vendor (BioIVT). The individuals were non-febrile and verbally screened to confirm no signs or symptoms of infection were present within 3 days prior to sample collection. They were also verbally screened to confirm that they were not currently undergoing antibiotic treatment and had not taken antibiotics within 3 days prior to sample collection. Further, all samples were shown to be negative for HIV, West Nile, Hepatitis B, and Hepatitis C by molecular or antibody-based testing. Samples were collected in PAXgene Blood RNA tubes and treated per the manufacturer's protocol. Samples were stored and transported at − 80 °C.

### Rapid isothermal assay

Our goal was to create a rapid assay, and isothermal reactions run much faster than traditional qPCR. Thus, loop-mediated isothermal gene expression (LAMP) assays were designed to span exon junctions, and at least three core (FIP/BIP/F3/B3) solutions meeting these design criteria were identified for each marker and evaluated for successful amplification of cDNA and exclusion of gDNA. Where available, loop primers (LF/LB) were subsequently identified for best core solutions to generate a complete primer set. Solutions were down-selected based on efficient amplification of cDNA and RNA, selectivity against gDNA, and the presence of single, homogenous melt peaks. The final primer sets are attached as Supplementary Table 2.

We designed an analytical validation panel of 61 blood samples from patients in multiple infection classes, including healthy, bacterial or viral. A subset of samples from patients with bacterial or viral infection came from patients with an infection that had progressed to sepsis. Whole blood samples were collected in PAXgene Blood RNA stabilization vacutainers, which preserve the integrity of the host mRNA expression profile at the time of draw. Total RNA was extracted from a 1.5 mL aliquot of each stabilized blood sample using a modified version of the Agencourt RNAdvance Blood kit and protocol. RNA was heat treated at 55 °C for 5 min then snap-cooled prior to quantitation. Total RNA material was distributed evenly across LAMP reactions measuring the five markers in triplicate. LAMP assays were carried out using a modified version of the protocol recommended by Optigene Ltd, and performed on a QuantStudio 6 Real-Time PCR System.

### Statistical analyses

Analyses were performed in R version 3 and Python version 3.6. The area under the receiver operating characteristic curve (AUROC) was chosen as the primary metric for model evaluation since it provides a general measure of diagnostic test quality without depending on prevalence or having to choose a specific cutoff point.

All validation dataset analyses use the locked 6-mRNA logistic regression output, i.e., predicted probabilities. AUROCs for additional markers (Table 3) are calculated from the available data for each marker. For the logistic regression model that includes the 6-mRNA predicted probabilities along with other markers as predictor variables, conditional multiple imputation was used for values to ensure model convergence. Since AUROC may fail to detect poor calibration on validation data (since subject rankings may still hold), we also demonstrated that a cutoff chosen from training data maintains good sensitivity and specificity in validation data even before recalibration. Due to the relatively small sample size, we made inter-group comparisons without assumptions of normality where possible (Kruskal–Wallis rank sum or Mann–Whitney *U* test). Medians and interquartile ranges are given for continuous variables.

### Ethics approval and consent to participate

COVID-19 blood samples were collected between March and April 2020. The studies were conducted following approvals for the collection of biomaterial for transcriptomic analysis for patients with lower respiratory tract infections provided by the Ethics Committees of the participating hospitals. The studies were conducted under the 23/12.08.2019 approval of the Ethics Committee of Sotiria Athens General Hospital; and the 26.02.2019 approval of the Ethics Committee of ATTIKON University General Hospital. Written informed consent was provided by patients or by first-degree relatives in case of patients unable to consent. For Australia/WIMR study, the written consent is provided by all patients or by first-degree relatives in case of patients unable to consent. The study was approved by the human research ethics committee of the Nepean Blue Mountains Area Health Services. For PREVISE study, the IRB was approved by COMITE DE ETICA DE LA INVESTIGACION CON MEDICAMENTOS DEL AREA DE SALUD DE SALAMANCA, Paseo de San Vicente, 58-18, 237007 Salamanca, Spain. For healthy commercial controls, blood RNA tubes were prospectively collected from healthy controls (HC) through a commercial vendor (BioIVT) under IRB approval (Western IRB #2016165) using informed consent. Stanford ICU databank and PROMPT studies have previously been published and provided IRB details.

All necessary patient/participant consent has been obtained and the appropriate institutional forms have been archived.

### Consent for publication

All necessary patient/participant consent has been obtained and the appropriate institutional forms have been archived.

## Results

**Figure 2:**
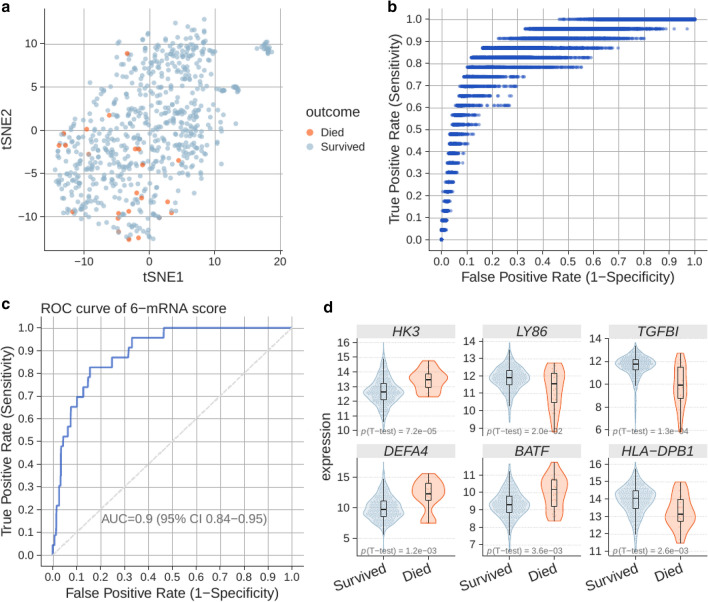
Training data for the 6-mRNA classifier. (**a**) Visualization of 705 samples across 21 studies in low dimension using t-SNE. (**b**) Logistic regression model selection. Each dot corresponds to a model defined by a combination of logistic regression hyperparameters and a decision threshold. Entire search space (100 hyperparameter configurations) is shown. (**c**) ROC plot for the best model. The plot is constructed using pooled probabilities from cross-validation folds. (**d**) Expression of the 6 genes used in the logistic regression model according to mortality outcomes.

We first identified 21 studies^25–40^ with 705 patients with viral infections (no patient with SARS-CoV-2) based on computer-aided adjudication and available outcomes data (see “Materials and methods”; Fig. 1 and Table 1). These studies included a broad spectrum of clinical, biological, and technical heterogeneity as they profiled blood samples from viral infections from 14 countries using mRNA profiling platforms from four manufacturers (Affymetrix, Agilent, Illumina, Nanostring). Within each dataset, the number of patients who died were very low (two or less for all but one study), meaning traditional approaches for biomarker discovery that rely on a single cohort with sufficient sample size would not have been effective. However, there were sufficient cases (23 deaths within 30 days of sample collection) across these 705 patients. Sample size analysis using pmsampsize package^41^ suggested minimal sample size of 450 patients with 18 cases, confirming the adequacy of the pooled dataset. Our previously described approaches for integrating independent datasets and leveraging heterogeneity allowed us to learn across the whole pooled dataset^19,42,43^. Visualization of the 705 conormalized samples using all genes present across the studies using t-stochastic neighbor embedding (t-SNE), showed that there was no clear separation between the samples from patients who died and those who survived (Fig. 2a).

### 6-mRNA logistic regression-based model accurately predicts viral patient mortality across multiple retrospective studies

In the pandemic, time to production of an assay was critical. We limited our test to no more than 6 genes because (1) it reduces R&D time and (2) it allows for higher throughput, since no more than 4–5 genes can be quantitated in a single well via qRT-PCR (and only 1 target per well for qRT-LAMP). In theory, a 30-gene classifier may perform slightly better, but if it does so at an expense of 6 extra months to market a fivefold reduction in throughput, it seems to us not to be worthwhile.

Nevertheless, to assess the impact of this limitation, we trained several classification models where the number of genes ranged from 1 to 30. Coincidentally, the model with 6 genes had the highest AUROC (Supplementary Fig. 1).

Next, across the machine learning algorithms employed in our analyses, best result was achieved using neural network classifier, followed by logistic regression (Supplementary Fig. 2). We chose logistic regression because it had competitive AUROC, and was least likely to overfit on previously unseen data. Further, within logistic regression models, those trained using random cross-validation were more accurate than those trained using other variants of cross-validation. Finally, within the different 6-mRNA logistic regression-based models trained using CV, the model with highest AUROC used the following 6 genes: *TGFBI, DEFA4, LY86, BATF, HK3* and *HLA-DPB1*. It had an AUROC of 0.896 (95% CI 0.844–0.949) (Figs. 2b,c; Supplementary Fig. 3). Each of the 6 genes were significantly differentially expressed between patients with viral infections who survived and those who did not, of which 3 genes (*DEFA4, BATF, HK3*) were higher and 3 genes (*TGFBI, LY86, HLA-DPB1*) were lower in those who died (Fig. 2d). Based on the cross-validation, the 6-mRNA logistic regression model had a 91% sensitivity and 68% specificity for distinguishing patients with viral infection who died from those who survived. We used this model, referred to as the 6-mRNA classifier, as-is for validation in multiple independent retropective cohorts and a prospective cohort.

### 6-mRNA classifier is an age-independent predictor of mortality in patients with viral infections

Age is a known significant predictor of 30-day mortality in patients with respiratory viral infections. To assess the added value of the new prognostic information of the 6-mRNA classifier with regards to age in the training data, we fit a binary logistic regression model with age and pooled cross-validation 6-mRNA classifier probabilities as independent variables. The 6-mRNA score was significantly associated with increased risk of 30-day mortality (P < 0.001), but age was not (P = 0.06).

### Validation of the 6-mRNA classifier in multiple independent retrospective cohorts

**Figure 3:**
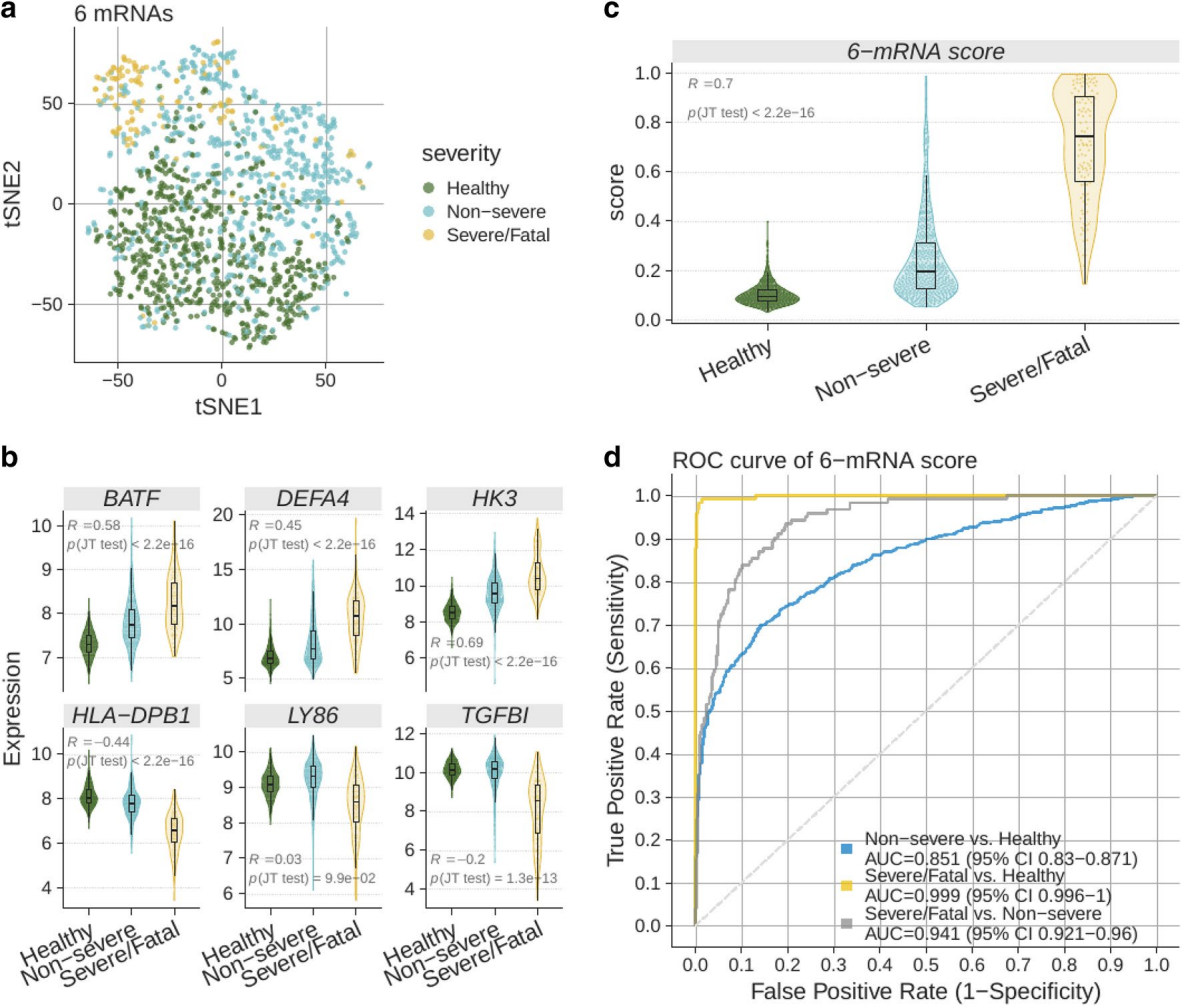
Validation of the 6-mRNA classifier in the independent retrospective non-COVID-19 cohorts. (**a**) Visualization of the samples using t-SNE. (**b**) Expression of the 6 genes used in the logistic regression model in patients with clinically relevant subgroups. (**c**) 6-mRNA classifier accurately distinguishes non-severe and severe patients with COVID-19 as well as those who died. (**d**) ROC plot for the subgroups. Excluding healthy samples, about 65% of the blood samples were estimated to have been collected “early”, and 35% “late”. See “Standardized severity assignment for retrospective non-COVID-19 patient samples” for details.

We applied the locked 6-mRNA classifier to 1417 transcriptome profiles of blood samples across 21 independent cohorts from patients with viral infections (663 healthy controls, 630 non-severe, 124 severe/fatal) in 10 countries (Supplementary Table 1). Visualization of the 1417 samples using expression of the 6 genes showed patients with severe outcome clustered closer (Fig. 3a). Among the 6 genes, over-expressed genes (*HK3, DEFA4, BATF*) were positively correlated with severity of viral infection, and under-expressed genes (*HLA-DPB1, LY86, TGFBI*) were negatively correlated with severity (Fig. 3b). Importantly, the 6-mRNA classifier score was positively correlated with severity and was significantly higher in patients with severe or fatal viral infection than those with non-severe viral infections or healthy controls (Fig. 3c). Finally, the 6-mRNA classifier score distinguished patients with severe viral infection from those with non-severe viral infection (AUROC = 0.941, 95% CI 0.921–0.96) and healthy controls (AUROC = 0.999, 95% CI 0.996–1) (Fig. 3d).

We plotted ROC curves to assess the discriminative ability of the 6-mRNA classifier among the following subgroups of clinical interest: healthy controls, non-severe cases, severe, and fatal outcomes (Fig. 3d). Healthy controls are presented (though not mixed with non-severe viral infections in comparison) since some viral infections such as COVID-19 can be asymptomatic. All pairwise comparisons showed robust performance of the classifier on the independent data, achieving AUROC point-estimates between 0.86 (non-severe vs. healthy) and 1 (severe vs. healthy).

### Validation of the 6-mRNA logistic regression model in two independent COVID-19 cohorts

**Figure 4:**
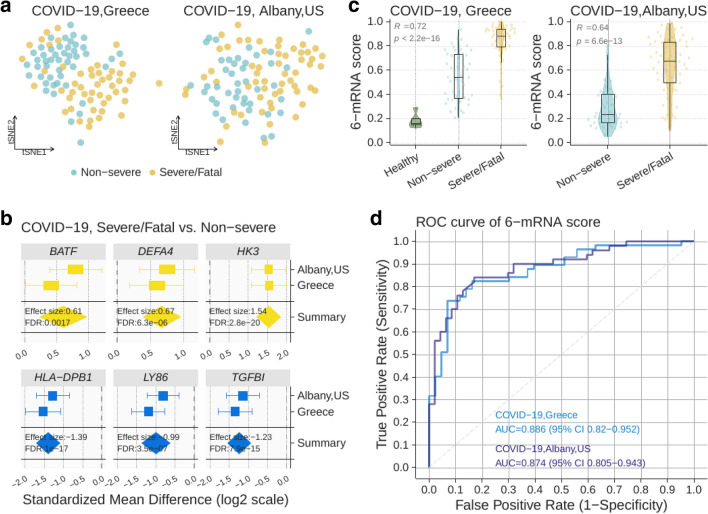
Validation of the 6-mRNA classifier in the COVID-19 cohorts. (**a**) Visualization of prospective (Greece, N = 97) and retrospective (Albany, US, N = 100) samples in the independent validation cohorts using t-SNE. (**b**) Expression of the 6 genes used in the logistic regression model in patients with severe/fatal and non-severe SARS-CoV-2 viral infection. (**c**) 6-mRNA classifier accurately distinguishes non-severe and severe patients with COVID-19 as well as those who died. (**d**) ROC plot for non-severe COVID-19 vs. severe or death (samples from healthy controls not included).

We further validated the 6-mRNA logistic regression model in two independent cohorts of patients with COVID-19. In one of the cohorts, we prospectively enrolled 97 adult patients with pneumonia by SARS-CoV-2 in Greece (Greece cohort). There were 47 patients with non-severe COVID-19 disease, whereas 50 had severe COVID-19, of which 16 died (Table 2). We also used gene expression data for 100 COVID-19-positive participants in the GSE157103 study in Gene Expression Omnibus database (Albany cohort). There were 43 patients with non-severe COVID-19 disease and 57 with severe COVID-19. Visualization of both cohorts in low dimension using expression of the 6 mRNAs (without the classifier) revealed a degree of separation between patients with severe COVID-19 disease and those with non-severe disease (Fig. 4a). When comparing expression of the 6 mRNAs in patients with non-severe COVID-19 disease to those with severe disease, expression of each changed statistically significant in the same direction as the training data in both cohorts (P < 0.05) (Fig. 4b).

**Table 2:** Demographics, severity scores, and severity markers for the prospective COVID-19 cohort, overall and split by mortality. P-values correspond to Mann–Whitney tests for difference of means and chi-square tests for difference of proportions between the survival and mortality groups. Unless indicated otherwise, numbers shown are median [IQR].

Variable	Overall	Death	Survival	P value
N	97	16	81	
Age (years)	62 [52, 72.25]	68.50 [62.75, 84.25]	60.00 [50.75, 70.25]	0.003
Gender = male (%)	68 (70.1)	12 (75.0)	56 (69.1)	0.865
White blood cells (/mm^3^)	6770 [5145, 10,227.50]	8540.00 [5542.50, 12,510.00]	6480.00 [5145.00, 9622.50]	0.275
Neutrophils (%)	78.10 [68.35, 86.60]	88.95 [86.40, 93.03]	77.09 [65.22, 83.75]	< 0.001
Lymphocytes (%)	12.70 [7.20, 21.15]	6.70 [3.65, 9.65]	14.03 [9.00, 22.42]	< 0.001
Platelets (/mm^3^)	215,000 [172,900, 266,000]	249,050 [180,750, 298,000]	214,000 [172,600, 260,800]	0.176
D-dimer (ng/ml)	977.90 [476.25, 2560.00]	4480.00 [2440.00, 13,161.50]	850.00 [437.50, 1947.50]	< 0.001
CRP (mg/l)	107.00 [31.60, 222.50]	224.75 [142.89, 260.75]	79.10 [28.80, 202.00]	0.002
SOFA score	3.00 [1.00, 6.00]	5.50 [4.00, 6.25]	2 [1, 6]	0.006
APACHE II	7.00 [5.00, 11.00]	11.00 [8.00, 13.50]	7 [4, 9]	0.001
Length of hospital stay	13.00 [11.00, 20.00]	13 [8.75, 17.25]	13 [11, 20]	0.410
Severe respiratory failure (%)	50 (51.5)	16 (100.0)	34 (42.0)	< 0.001

We applied the locked 6-mRNA classifier to the 97 COVID-19 patients and the 5 healthy controls in the Greece cohort. The 6-mRNA score was correlated with severity in both cohorts (Greece cohort: R = 0.72, P < 2.2e−16; Albany cohort: R = 0.64, P = 6.6e−13) (Fig. 4c). In particular, the model distinguished patients with severe respiratory failure from non-severe patients with an AUROC of 0.89 (95% CI 0.82–0.95) in the Greece cohort, and 0.87 (95% CI 0.80–0.94) in the Albany cohort (Fig. 4d).

We also assessed whether the 6-mRNA score is an independent predictor of severity in patients with COVID-19 by including other predictors of severity (age, SOFA score, CRP, PCT, lactate, and gender) in a logistic regression model. As expected, due to small sample size, and correlations between markers, no markers except SOFA were statistically significant predictors of severe respiratory failure (Supplementary Table 3).

For clinical applications, AUROC is a more relevant indicator of marker performance. To that end, we compared the 6-mRNA score to other clinical parameters of severity using AUROC (Table 3 and Supplementary Figs. 4, 5). The 6-mRNA score was the most accurate predictor of severe respiratory failure and death except SOFA. The AUROC confidence intervals were overlapping because the study was not powered to detect statistically significant differences. Of note, the 6-mRNA score was significantly more accurate for predicting severe respiratory failure and death than the only assay under the FDA Emergency Use Authorization, the IL-6. As a proxy for assessing how the 6-mRNA score might add to a clinician’s bedside severity assessment, we evaluated whether a combination of our classifier with the SOFA score improves over SOFA alone for the prediction of severe respiratory failure. The two scores together had an AUROC of 0.95; the continuous net reclassification improvement (cNRI) was 0.43 [95% CI 0.04–0.81, P = 0.03]. Together, these results suggest a potential improvement in clinical risk prediction when adding the 6-mRNA score to standard risk predictors; but definitive conclusion requires validation in additional independent data.

**Table 3:** Prognostic power of the 6-mRNA signature classifier and comparator scores and markers in the independent prospective COVID-19 cohort. Shown are AUROCs for non-missing data, plus 95% CI. The fourth column is a ‘fair’ assessment of the 6-mRNA signature classifier, i.e. the performance on the subset of patients that was available to the comparator.

Comparator marker	Number available	Comparator AUROC	6-mRNA classifier AUROC	P value
**(a) Prognostic power for predicting severe respiratory failure. Bold font indicates predictor with higher AUROC, which in nearly all cases is the 6-mRNA classifier. The P value column corresponds to DeLong test for difference between paired ROC curves**				
6-mRNA classifier	97		0.89 (0.82–0.95)	
SOFA	96	**0.93**	0.89	0.31
APACHE II	93	0.83	**0.89**	0.19
Age	96	0.78	**0.89**	0.07
PCT	76	0.80	**0.89**	0.07
CRP	97	0.86	**0.89**	0.58
Lactate	45	0.75	**0.82**	0.52
IL-6	97	0.73	**0.89**	< 1e−3
suPAR	97	0.79	**0.89**	0.04
**(b) Prognostic power for predicting mortality. Bold font indicates predictor with the higher AUROC. The P value column corresponds to DeLong test for difference between paired ROC curves**				
6-mRNA classifier	97		0.78 (0.64–0.92)	
SOFA	96	0.72	**0.78**	0.43
APACHE II	93	0.76	**0.77**	0.84
Age	96	0.74	**0.78**	0.68
PCT	76	0.73	**0.77**	0.63
CRP	97	0.74	**0.78**	0.62
Lactate	45	0.78	**0.80**	0.84
IL-6	97	0.57	**0.78**	0.003
suPAR	97	0.74	**0.78**	0.71

### Performance by age group

To evaluate the performance of the 6-mRNA score by age group we compared results in the non-COVID-19 independent retrospective data in children under 12 years of age (677 samples) and adults over 18 years of age (740 samples). The results are shown in Supplementary Fig. 6. We concluded that although the classifier was developed using only adult samples, it performed with similar accuracy in children with non-COVID-19 viral infections.

### Pooled results

**Figure 5:**
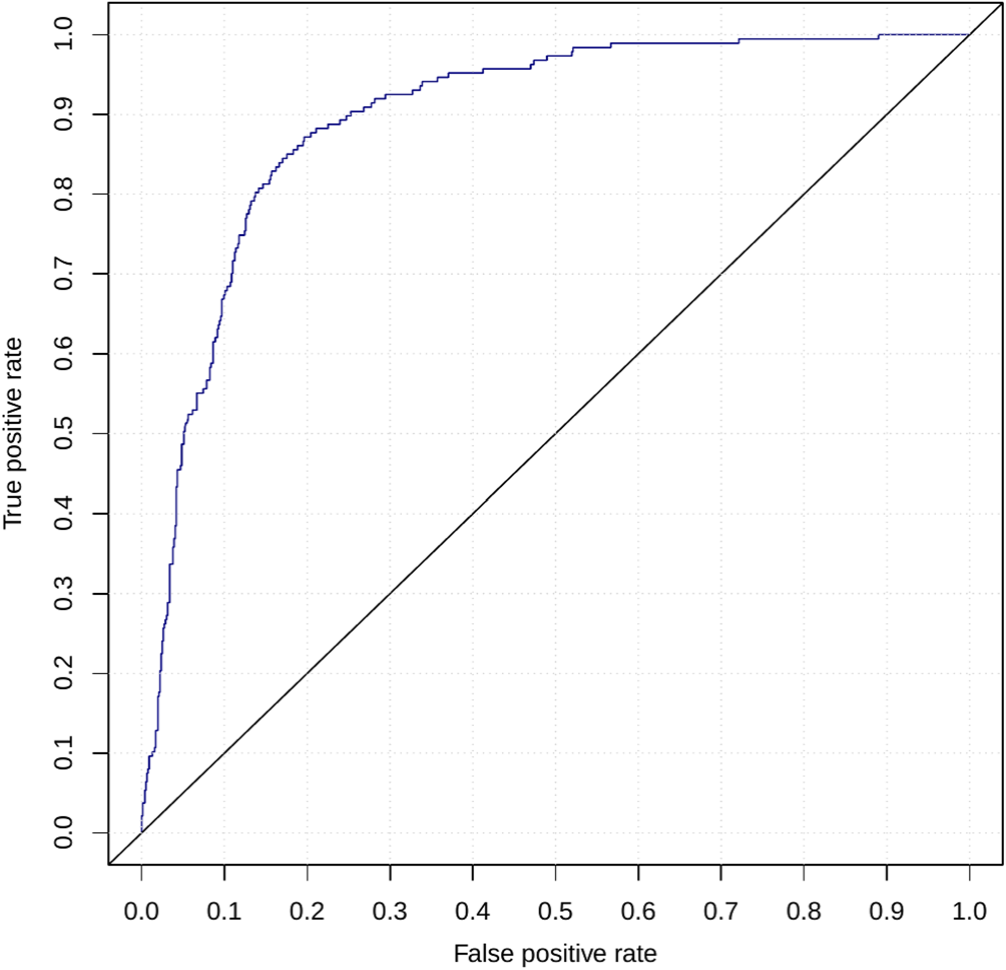
Validation of the 6-mRNA classifier in the COVID-19 and non-COVID-19 cohorts (pooled results of data in Figs. 3, 4, excluding healthy subjects). The total number of samples was 951. The number of cases (severe, including fatal, disease) was 187. The remaining samples (764) had non-severe disease. AUROC = 0.90 (95% CI 0.87–0.92).

We combined the predicted probabilities from the COVID-19 and non-COVID-19 independent cohorts and plotted the corresponding ROC graph (Fig. 5). The corresponding AUROC was 0.90 (95% CI 0.87–0.92).

### Translation to a clinical report

To improve utility and adoption, a risk prediction score should be presented to clinicians in an intuitive and actionable test report. To that end, we discretized the 6-mRNA score in three bands: low-risk, intermediate-risk, and high-risk of severe outcome. The performance characteristics of each band are shown in Table 4. The table shows performance of the test on retrospective data (excluding healthy controls) using two versions of decision thresholds: thresholds optimized on the training data (Table 4a), and thresholds optimized using the retrospective test set (Table 4b). The outcome was severe infection. Tables 4c,d show corresponding results on the COVID-19 data, using severe respiratory failure as outcome.

**Table 4:** Test characteristics of the 6-mRNA score in non-COVID-19 and COVID-19 patients using the three-band test report. “Severe in band” is the number of patients with severe viral infection assigned to the corresponding band. “Non-severe in band” is the number of patients with non-severe viral infection assigned to the corresponding band. The “Percent severe in band” is the percentage of patients in the band who had severe outcome. The “In-band” column is the percentage of patients assigned by the classifier to the corresponding band.

Band	Severe in band	Non-severe in band	Percent severe in band (%)	Sensitivity (%)	Specificity (%)	Likelihood ratio	In-band (%)
**(a) Non-COVID-19 results. The band thresholds were set using training data and locked**							
Low risk	12	570	2.0	85	85	0.18	77
Intermediate risk	43	78	36	54	88	4.6	16
High risk	25	26	49	31	96	8.1	6.7
**(b) Non-COVID-19 results. The band thresholds were set using the retrospective data**							
Low risk	2	420	0.47	98	62	0.04	56
Intermediate risk	68	246	22	85	64	2.3	42
High risk	10	8	56	12	99	11	2.4
**(c) Prospective COVID-19 results. The band thresholds were set using training data and locked**							
Low risk	2	18	10	96	38	0.10	21
Intermediate risk	20	28	42	40	40	0.67	50
High risk	28	1	97	56	98	26	30
**(d) Prospective COVID-19 results. The band thresholds were set using the prospective data**							
Low risk	0	12	0	100	26	0.0	12
Intermediate risk	17	32	35	34	32	0.50	51
High risk	33	3	92	66	94	10	37
**(e) Retrospective COVID-19 results. The band thresholds were set using training data and locked**							
Low risk	14	37	27	75	86	0.28	51
Intermediate risk	33	6	85	58	86	4.1	39
High risk	10	0	100	18	100	Inf	10
**(f) Retrospective COVID-19 results. The band thresholds were set using the retrospective data**							
Low risk	1	16	6	98	37	0.047	17
Intermediate risk	14	24	37	25	44	0.44	38
High risk	42	3	93	74	93	11	45

### Translation to a rapid assay

Any risk prediction score should be rapid enough to fit into clinical workflows. We thus developed a LAMP assay as a proof of concept for a rapid 6-mRNA test. We further showed that across 61 clinical samples from healthy controls and acute infections of varying severities that the LAMP 6-mRNA score and the reference NanoString 6-mRNA score had very high correlation (r = 0.95; Supplementary Fig. 7). These results demonstrate that with further optimization the 6-mRNA model could be translated into a clinical assay to run in less than 30 min.

### Impact of computer-aided adjudication

To assess the impact of adding computer-adjudicated samples to the training set, we trained another classifier using only the clinically-adjudicated infections. The training set contained only patients which were clinically adjudicated to have viral infection. The total number of cases was 14, compared with 23 in the computer-adjudicated training set. We hypothesized that due to the smaller number of samples, the classifier trained on this set would have larger bias, which we defined as the performance difference between the cross-validation training AUROC and AUROC on the independent data. The bias for the locked classifier was 0, whereas the bias for the classifier trained on the clinically-adjudicated data was 0.017. The complete results of this analysis are shown in Supplementary Table 4 and Supplementary Fig. 8. Note that this is a counter-factual analysis because the choice of computer-aided adjudication had to be made before locking the classifier and applying it to independent data. Given this, we reasoned during development that it would be too risky to use clinically-adjudicated training data because it had only 14 cases. Hence we decided to use computer-aided adjudication.

## Discussion

The severe economic and societal cost of the ongoing COVID-19 pandemic, the fourth viral pandemic since 2009, has underscored the urgent need for a prognostic test that can help stratify patients as to who can safely convalesce at home in isolation and who needs to be monitored closely. Here we integrated 705 peripheral blood transcriptome profiles across 21 heterogeneous studies from patients with viral infections, none of whom were infected with SARS-CoV-2. Despite the substantial biological, clinical, and technical heterogeneity across these studies, we identified a 6-mRNA host-response signature that distinguished patients with severe viral infections from those without. We demonstrated generalizability of this 6-mRNA model first in a set of 21 independent heterogeneous cohorts of 1417 retrospectively profiled samples, and then in two independent retrospectively and prospectively collected cohorts of patients with SARS-CoV-2 infection, in United States and Greece, respectively. In each validation analysis, the 6-mRNA classifier accurately distinguished patients with severe outcome from those with non-severe outcomes, irrespective of the infecting virus, including SAR-CoV-2. Importantly, across each analysis, the 6-mRNA classifier had similar accuracy, measured by AUROC, demonstrating its generalizability and robustness to biological, clinical, and technical heterogeneity. Although this study was focused on development of a clinical tool, not a description of transcriptome-wide changes, the applicability of the signature across viral infections further demonstrates that host factors associated with severe outcomes are conserved across viral infections, which is in line with our recent large-scale analysis^20^.

While many risk-stratification scores and biomarkers exist, few are focused specifically on viral infections. Of the recent models specifically designed for COVID-19, most are trained and validated in the same homogenous cohorts, and their generalizability to other viruses is unknown because they have not been tested across other viral infections^14^. Consequently, when a new virus, such as SARS-CoV-2, emerges, their utility is substantially limited. However, we have repeatedly demonstrated that the host response to viral infections is conserved and distinct from the host response to other acute conditions^15–20^.

Here, building upon our prior results, we developed a 6-mRNA classifier specifically trained in patients with viral infection to risk stratify better than other existing biomarkers. Further, the only assay authorized for clinical use in risk-stratifying COVID-19 (IL-6 measured in blood), substantially underperformed our proposed 6-mRNA model here. That said, the nominal improvement over existing biomarkers (Table 3) for prediction of severe respiratory failure requires larger cohorts to confirm statistical significance. The 6-mRNA score is nominally worse than SOFA, but SOFA requires 24 h to calculate, while the 6-mRNA score could be run in 30 min, demonstrating its utility as a triage test. The synergy (positive NRI) in combination with SOFA also suggests that the 6-mRNA score could improve practice in combination with clinical gestalt. The 6-mRNA score has been reduced to practice as a rapid isothermal quantitative RT-LAMP assay, suggesting that it may be practical to implement in the clinic with further development.

Our goal in this study was not to investigate underlying biological mechanisms, but to address the urgent need for a prognostic test in SARS-CoV-2 pandemic, and to improve our preparedness for future pandemics. However, using immunoStates database (https://metasignature.khatrilab.stanford.edu)^44^, we found 5 out of the 6 genes (*HK3, DEFA4*, *TGFBI, LY86, HLA-DPB1*) are highly expressed in myeloid cells, including monocytes, myeloid dendritic cells, and granulocytes. This is in line with our recent results demonstrating that myeloid cells are the primary source of conserved host response to viral infection^20^. Further, we have previously found that *DEFA4* is over-expressed in patients with dengue virus infection who progress to severe infection^45^, and in those with higher risk of mortality in patients with sepsis^18^. *HLA-DPB1* belongs to the HLA class II beta chain paralogues, and plays a central role in the immune system by presenting peptides derived from extracellular proteins. Class II molecules are expressed in antigen presenting cells (B lymphocytes, dendritic cells, macrophages). Reduced expression of *HLA-DPB1* described herein is fully compatible with the decreased expression of HLA-DR on the cell membranes of circulating monocytes of patients with severe respiratory failure by SARS-CoV-2. This is a unique immune dysregulation where despite the down-regulation of HLA-DR monocytes remain potent for the production of pro-inflammatory cytokines, namely TNFα and IL-6. This complex immune dysregulation fully differentiates critically ill patients with COVID-19 from patients with bacterial sepsis^46^ in patients with severe outcome and suggests dysfunctional antigen presentation that should be further investigated. Similarly, *BATF* is significantly over-expressed, and *TGFBI* is significantly under-expressed in patients with sepsis compared to those with systemic inflammatory response syndrome (SIRS)^15^. Finally, lower expression of *TGFBI* and *LY86* in peripheral blood is associated with increased risk of mortality in patients with sepsis^18^. These results further suggest that there may be a common underlying host immune response associated with severe outcome in infections, irrespective of bacterial or viral infection. Consistent differential expression of these genes in patients with a severe infectious disease across heterogeneous datasets lend further support to our hypothesis that dysregulation in host response can be leveraged to stratify patients in high- and low-risk groups.

Our study has several limitations. First, our study uses retrospective data with large amount of heterogeneity for discovery of the 6-mRNA signature; such heterogeneity could hide unknown confounders in classifier development. However, our successful representation of biological, clinical, and technical heterogeneity also increased the a priori odds of identifying a parsimonious set of generalizable prognostic biomarkers suitable for clinical translation as a point-of-care. Second, owing to practical considerations for urgent need, we focused on a preselected panel of mRNAs. It is possible that similar analysis using the whole transcriptome data would find additional signatures, though with less clinical data. Third, a common limitation in all these types of pandemic observational studies is a lack of understanding of the effect of time from symptoms onset. Fourth, up to about 35% of the non-COVID-19 validation samples could have been sampled late in the disease, which may affect interpretation of the results. Finally, additional larger prospective cohorts are needed to further confirm the accuracy of the 6-mRNA model in distinguishing patients at high risk of progressing to severe outcomes from those who do not.

Overall, our results show that once translated into a rapid assay and validated in larger prospective cohorts, this 6-mRNA prognostic score could be used as a clinical tool to help triage patients after diagnosis with SARS-CoV-2 or other viral infections such as influenza. Improved triage could reduce morbidity and mortality while allocating resources more effectively. By identifying patients at high risk to develop severe viral infection, i.e., the group of patients with viral infection who will benefit the most from close observation and antiviral therapy, our 6-mRNA signature can also guide patient selection and possibly endpoint measurements in clinical trials aimed at evaluating emerging anti-viral therapies. This is particularly important in the setting of current COVID-19 pandemic, but also useful in future pandemics or even seasonal influenza.

## Conclusions

With further study, the classifier could assist in the risk assessment of COVID-19 and other acute viral infections patients to determine severity and level of care, thereby improving patient management and reducing healthcare burden.

## Supplementary Information

Supplementary Information.

## Data Availability

The public cohorts are available under their respective study IDs. The Stanford ICU Databank study is available at 10.1038/s41467-020-14975-w. The COVID-19 NanoString data are available upon reasonable request.

## Abbreviations

COVID 19: Coronavirus disease 2019
ARDS: Acute respiratory distress syndrome
CD3: Cluster of differentiation 3
CD4: Cluster of differentiation 4
ML: Machine learning
IMX-BVN-2: Inflammatix Bacterial-Viral-Noninfected classifier version 2
PBMC: Peripheral blood mononuclear cells
ED: Emergency department
ICU: Intensive care unit
SOFA: Sequential organ failure assessment
HIV: Human immunodeficiency virus
cDNA: Complementary DNA
gDNA: Genomic DNA
LAMP: Loop-mediated isothermal amplification
PCR: Polymerase chain reaction
CI: Confidence interval
FDA: US Food and Drug Administration
cNRI: Continuous net reclassification index
